# Recurrent Transient Ischemic Attacks in a Patient with Multiple Pacemaker Leads

**DOI:** 10.19102/icrm.2023.14042

**Published:** 2023-04-15

**Authors:** Thinzar Shwe, Aneeqa Javed, Ravi Patel, Philippe Akhrass

**Affiliations:** ^1^Cardiology Department, Staten Island University Hospital, New York, NY, USA; ^2^Internal Medicine Department, Staten Island University Hospital, New York, NY, USA

**Keywords:** Pacemaker leads, permanent pacemaker, superior vena cava syndrome, transient ischemic attack

## Abstract

Venous complications—specifically, stenosis and thrombosis—are both well-known complications of transvenous implantation of pacemakers and defibrillators. Although they are a well-recognized phenomenon, these complications are rarely of clinical significance. One of the most concerning complications is the development of superior vena cava (SVC) syndrome. Studies have found that the incidence of SVC syndrome varies from 1 in 3,100 to 1 in 650 patients. The azygos–hemiazygos venous system is the most commonly observed collateral. We report a case of a 71-year-old female patient who presented with stroke-like symptoms during the injection of agitated saline bubbles while performing an echo and was found to have an unusual venous collateral circulation formed as a result of brachiocephalic and SVC obstruction from multiple pacemaker leads. Our patient’s clinical presentation was extremely unique, and we did not find any cases during our literature search reporting a similar presentation. Multiple collaterals formed between the brachiocephalic and subclavian veins, and bilateral pulmonary veins in our patient allowed the injected air bubbles from the venous system to reach the left side of the heart and eventually the cerebrovascular system, resulting in these transient ischemic attacks. These attacks eventually resolved as the air bubbles were dissolved and washed away by the continuous blood flow. It is advisable to monitor the patient for possible venous stenosis and SVC syndrome after any device insertion during regular device follow-up appointments.

## Introduction

Venous complications—specifically, stenosis and thrombosis—are both well-known complications of transvenous pacemaker and defibrillator implantation.^1,2^ Studies analyzing the risk factors associated with venous thrombosis and stenosis related to pacemaker placement have been inconclusive so far.^3^ Although they are a well-recognized phenomenon, complications are rarely of clinical significance. One of the most concerning complications is the development of superior vena cava (SVC) syndrome. Studies have found that the incidence of SVC syndrome varies from 1 in 3,100 to 1 in 650 patients.^4–8^ The azygos–hemiazygos venous system is the most commonly observed collateral, with other routes consisting of the internal mammary veins, lateral thoracic veins, and vertebral veins.^9^ In a rare and unusual pathway, these collaterals may drain into the pulmonary veins or directly into the left side of the heart, forming a right-to-left shunt and leaving the patient susceptible to stroke, brain abscess, and other complications.

We report the case of a 71-year-old female patient who presented with stroke-like symptoms and was found to have an unusual venous collateral circulation formed as a result of brachiocephalic and SVC obstruction from multiple pacemaker leads.

## Case presentation

A 71-year-old female patient with a past medical history of recurrent transient ischemic attacks (TIAs) and antiphospholipid syndrome (on warfarin) was transferred from the outpatient nuclear imaging center to the emergency department for acute left-sided numbness and weakness after injection with tagged blood through a left peripheral intravenous access site. She was also known to have complete heart block requiring dual-chamber pacing for the previous 22 years. During that time, she underwent placement of multiple leads, a pocket revision, and a generator change.

**Figure 1:**
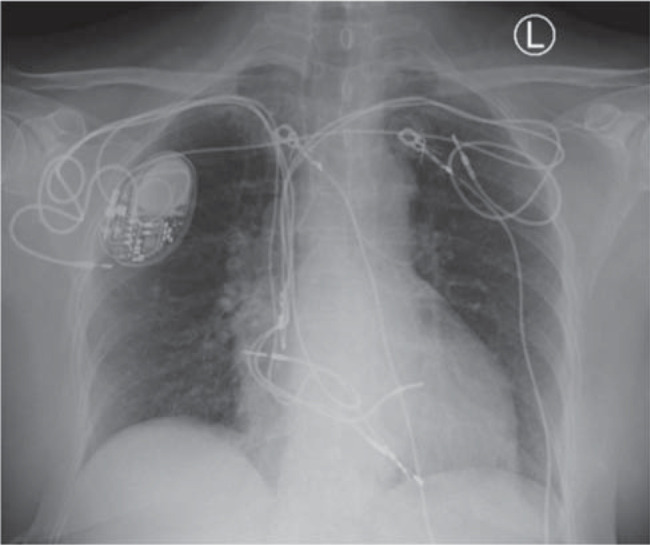
Chest X-ray showing right-sided pacemaker with 3 right-sided leads and 2 left-sided leads, including 1 tunneled across the chest over to the device on the right; also, note that a total of 5 leads pass through the superior vena cava.

The patient had been experiencing pain and discomfort over the pacemaker site for the past month with leukocytosis and, therefore, was sent for a nuclear medicine white blood cell scan to identify whether there was a potential pocket infection or another possible source of infection. Her U.S. National Institutes of Health Stroke Scale score^1^ was calculated to be 4 points. The patient was not a candidate for tissue plasminogen activator therapy. An emergent computerized tomography (CT) scan without contrast and a CT angiography scan of the head and neck were done to evaluate acute ischemic stroke and neurovascular patency, both of which were negative for acute intracranial pathology and vasculature compromise. Magnetic resonance imaging (MRI) of the head was further recommended by neurology but was not performed because the patient had multiple abandoned pacemaker leads (as seen in **Figure 1**), which were considered a contraindication to MRI. The patient’s symptoms steadily resolved over the course of a few hours on the same day.

**Figure 2:**
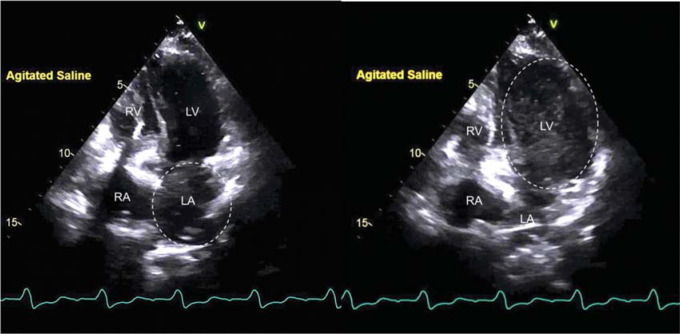
Apical 4-chamber transthoracic echocardiogram showing how agitated saline contrast bubbles initially appeared in the LA (as shown by the dashed circle) on the left image, followed by the left ventricle (shown by the dashed oval) on the right image. In normal anatomy, the bubbles will appear in the right atrium first, followed by the right ventricle right after injection. *Abbreviations:* RA, right atrium; RV, right ventricle.

Transthoracic echocardiography with agitated saline contrast was performed on day 2 of hospitalization to evaluate cardiac structural abnormalities. The patient had another episode of TIA while she was injected with agitated saline contrast from the right upper extremity at the end of the study. The study showed normal left ventricular systolic function without major valvular abnormalities. No apparent shunts were visualized through either the interatrial septum or ventricular septum on color Doppler. However, agitated saline bubbles initially appeared in the left atrium followed by the left ventricle before reaching the right side of the heart, as shown in **Figure 2**, which commonly suggests the presence of persistent left-sided SVC with unroofed coronary sinus.

**Figure 3:**
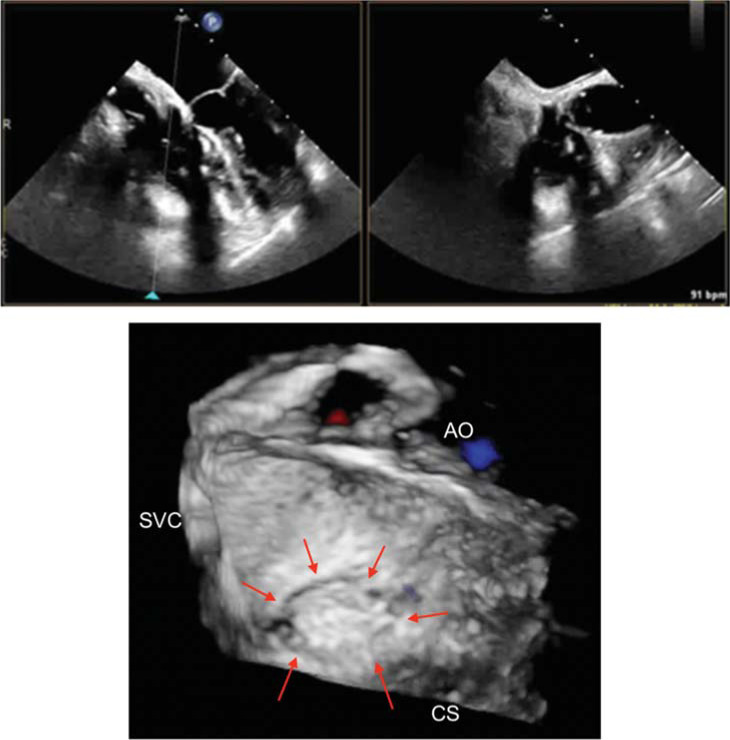
On top, biplane images can be seen. On bottom is a 3-dimensional transthoracic echocardiogram with color flow Doppler of the intact intra-atrial septum (red arrows surrounding the fossa ovalis) without evidence of shunt at the interatrial level. *Abbreviations:* AO, aorta; CS, coronary sinus; SVC, superior vena cava.

**Figure 4:**
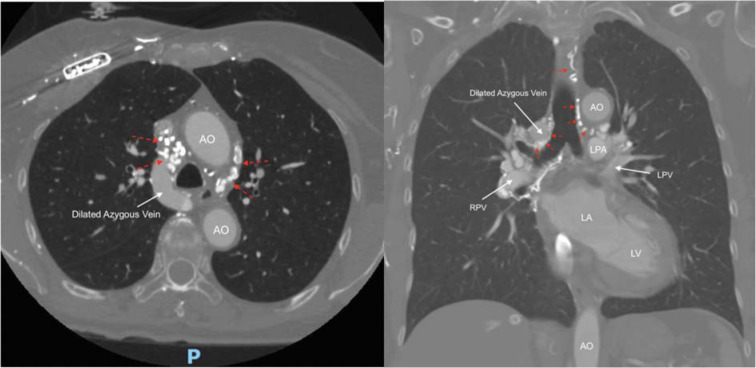
From left to right, axial and coronal segments of computed tomography angiography imaging of the heart; dashed red arrows indicate the network of collaterals directed toward bilateral pulmonary veins that had developed extensively across the chest. *Abbreviations:* AO, aorta; LA, left atrium; LPA, left pulmonary artery; LPV, Left pulmonary vein; LV, left ventricle; RPV, right pulmonary vein.

Transesophageal echocardiogram was performed on day 3 of the hospitalization to further evaluate the cardiac shunt, which revealed pacemaker leads in the right ventricle (RV) without any evidence of thrombus or vegetation. No evidence of transseptal shunt or defect was visualized on the 3-dimensional image of the interatrial septum in color Doppler mode, as shown in **Figure 3**. Therefore, extracardiac shunting from right to left was suspected. The pulmonary blood flow (Qp) to systemic blood flow (Qs) ratio (Qp/Qs) was calculated to be 0.9 using the continuity equation, which was not significant enough to cause cyanosis. Due to the patient’s recurrent stroke-like symptoms following intravenous injection, a decision was made to perform structural CT angiography of the coronary and chest vasculature on day 6 of her hospitalization. Health care professionals involved in the patient’s care were advised to avoid administering anything intravenously through the bilateral upper extremities, to de-air the system carefully, and to use an intravenous in-line filter if necessary. The CT angiography scan of the heart and coronaries with contrast showed multiple pacemaker leads passing through the SVC and terminating within the right atrium (RA) and RV. The right brachiocephalic vein was not visualized and therefore was assumed to be completely occluded, while the left brachiocephalic vein appeared patent. This study did not show a persistent left SVC but rather showed several veno-venous collaterals arising from the bilateral brachiocephalic veins/SVC draining into the bilateral pulmonary veins, as seen in **Figure 4**.

Findings from the structural CT scan of the cardiac vasculature were discussed with the patient, and a detailed history was obtained regarding her permanent pacemaker (PPM) leads. The patient received her first dual-chamber pacemaker with 2 leads placed on her left chest in 1995. In 2005, she had received a pacemaker with 2 new leads placed on the right side, while the 2 leads on her left were abandoned at the time. The patient also reported experiencing TIAs in 2006, 2009, and March 2011. She remained on aspirin and clopidogrel for these TIAs until late 2011, when she was found to have a positive antiphospholipid antibody result and was subsequently started on warfarin. Then, later in 2011, she was found to have a fractured RA lead and underwent pacemaker replacement. At the time, the fractured RA lead was not extracted due to the risk of damage to the working RV lead. In 2016, a new RV lead was placed as the existing ventricular lead had failed.

**Figure 5:**
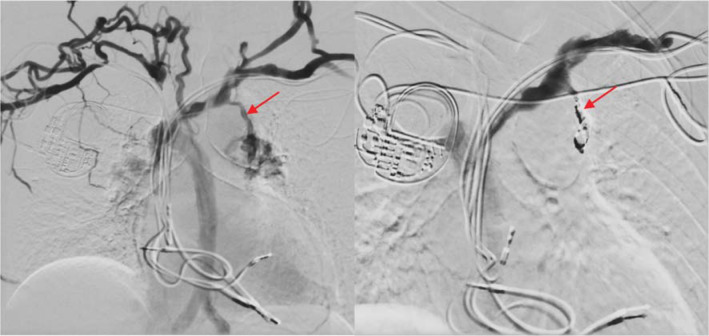
From left, a venogram from simultaneous upper-extremity injection of contrast dye shows the existence of a major collateral from the left brachiocephalic vein to the left pulmonary vein highlighted by a red arrow, which is subsequently coiled on the right image.

Bilateral upper extremity and central venograms were planned together with potential coil embolization of the venous collaterals. A central venogram was performed by simultaneously injecting intravenous contrast through the bilateral upper extremities on day 9 of the hospitalization, which showed a nearly occluded SVC with multiple proximal collateralizations. There was also a reflux of contrast seen in the azygos vein with small collaterals arising and supplying the area of the right hilum. The largest collateral system identified arose from the left brachiocephalic vein and drained into the left pulmonary vein. It was successfully coil embolized, as shown in **Figure 5**.

**Figure 6:**
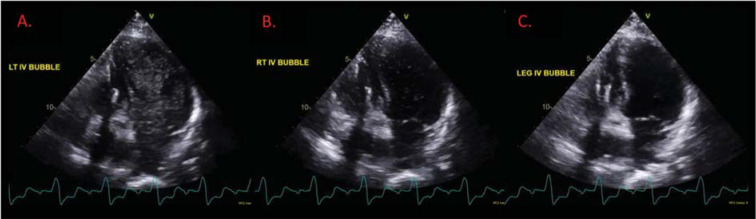
Follow-up transthoracic echocardiogram with agitated saline contrast injected from bilateral upper extremities and through the lower extremity to reassess shunting after coil embolization of a large collateral. **A:** A large number of agitated saline bubbles appeared in the left atrium and left ventricle before the right side with the left-sided injection in a manner suggestive of persistent extracardiac shunting. **B:** With the right upper-extremity injection, the density of the bubbles was reduced, following the same route in a manner suggestive of a lesser degree of shunt on the right compared to the left. **C:** An absence of agitated saline bubbles was seen with the lower-extremity injection through multiple cardiac cycles. *Abbreviations:* IV, intravenous; LT, left; RT, right; TTE, transthoracic echocardiogram.

Follow-up transthoracic echocardiography with agitated saline contrast through bilateral upper extremities and leg injection was performed to evaluate for residual shunting after coil embolization. As shown in **Figure 6**, despite successful coiling of the largest left collateral, there remained significant venous shunting to the left side of the heart with saline contrast injected from the left upper extremity.

It was collectively decided to avoid coiling the shunt further as there were concerns about potentially causing SVC syndrome by further impeding venous return from the upper half of the body (above the diaphragm) as well as the further formation of collaterals in the presence of marked SVC stenosis. The patient was discharged home on warfarin and her other home medications; additionally, she was advised to avoid intravenous injection through the upper extremities if possible and, if needed, to make sure the injection is de-aired carefully to prevent these TIAs.

## Discussion

Our patient’s clinical presentation was unique, and we did not find any cases during our literature search reporting a similar presentation. Our patient developed SVC obstruction because of the presence of multiple pacemaker leads; however, she did not develop SVC syndrome due to the development of adequate venous collaterals back to the systemic circulation. Consequently, the site and extent of her collateral venous flow resulted in a direct right-to-left shunt, which predisposed her to embolic events. Multiple collaterals formed between the brachiocephalic and subclavian veins and bilateral pulmonary veins in our patient, which allowed the injected air bubbles from the venous system to reach the left side of the heart and eventually the cerebrovascular system, resulting in TIAs. These attacks eventually resolved as the air bubbles were dissolved and washed away by the continuous blood flow.

Venous stenosis is a common complication of transvenous lead implantation.^10^ It is mostly asymptomatic due to the formation of collateral venous flow. At least 25% of patients with transvenous leads have some degree of SVC obstruction.^11^ SVC syndrome after pacemaker implantation is a closely related entity and occurs when the patient cannot develop venous collaterals on time. Although SVC syndrome associated with PPM implantation is rare, several cases have previously been reported in the literature.^11–14^ Acute venous obstruction is a result of acute thrombosis of the access vein, whereas late obstruction occurs due to stenosis of the vessel or obstruction of the collaterals.^15^

A study involving 212 patients who underwent device implantation reported that 61% of the patients developed various degrees of venous stenosis after receiving an implantable cardiac defibrillator or pacemaker and 26% had complete venous occlusion.^16^ This study also showed that the presence of venous occlusion correlates with the sum of lead diameters, the number of leads, and a history of prior procedures.^16^ Other studies show that the presence of multiple leads and a history of heart failure, tobacco smoking, arrhythmias, infections, or myocardial infarction increase the risk of venous obstruction after pacemaker implantation.^3,17^

In our case, the patient had a total of 5 leads (3 on the right and 2 on the left), which put her at high risk for venous stenosis. Furthermore, this patient had a positive antiphospholipid antibody result, which might have further increased the risk of venous stenosis/thrombosis. The mechanism of venous obstruction is not yet well understood but is thought to be due to the mechanical stress associated with the pacemaker leads.^14^ Abu-El-Haija et al. also hypothesized that venous stenosis could develop due to endothelial trauma occurring during the procedure.^16^ Multiple entries into the venous system during the procedure cause repetitive trauma to the endothelium, which may promote an inflammatory reaction that eventually leads to venous stenosis.^14^

Several studies looking at the prophylactic use of anticoagulants or antiplatelet medications to prevent the development of venous stenosis have offered conflicting results. Lelakowski et al. and Haghjoo et al. suggested that a patient on antiplatelet or anticoagulant therapy for other comorbidities had a protective effect against the development of access vein obstruction,^3,17^ whereas studies by Abu-El-Haija et al.^16^ and Goto et al.^18^ found no association between antiplatelet or anticoagulant use and the development of venous stenosis.

Looking at this patient’s history, her prior 3 TIA events before this hospitalization also had some involvement of intravenous injections with CT scans of the head not showing any infarcts. Whether these TIAs were related to a thromboembolic event or a hypercoagulable state due to antiphospholipid syndrome remains questionable as the patient had no other history of acute thrombotic events and currently has healthy children in their adulthood. These TIA events occurred 1 year after right-sided lead placement in 2005, which may suggest that the patient might have developed collaterals leading to embolic events, including her first TIA in 2006. In this case, the fact that our patient remained on warfarin for the past decade might have prevented a large thromboembolic event; however, it did not seem to have reduced the degree of venous stenosis and subsequent collateral formation.

Given our understanding of the potential complications of leaving multiple inactive leads in situ, this behooves practitioners to consider removing inactive leads at the time of new lead implantation. Despite various tools being available for lead extractions, they remain challenging and carry the risk for multiple major complications, including cardiac avulsion, vascular avulsion, pulmonary embolism, stroke, respiratory arrest or anesthesia-related complications, pacing system–related infection of a previously non-infected site, and death as well as minor complications as defined by the Heart Rhythm Society expert consensus.^19^ Multiple studies have documented procedural and clinical success rates of >90% in the removal of old leads at experienced centers.^20,21^ In the lead-related SVC syndrome, surgery, venoplasty, stenting, and thrombolytic therapy have been used as effective methods for managing SVC syndrome due to pacemaker leads.^1,22–24^

## Conclusion

In the United States alone, it is estimated that 400,000 devices are implanted each year, and 3 million people currently live nationwide with implanted cardiac devices.^25^ It is important to consider the long-term complications of these implanted devices and leads. Venous stasis and collateral formation in patients with multiple cardiac implantable electronic device leads are commonly subclinical; however, these devices may cause unusual presentations, such as recurrent TIA from left-to-right shunting, as we have described here. In the case of recurrent unexplained cerebrovascular accidents, a detailed evaluation may be warranted to identify a rare cause.
